# How Characters Are Learned Leaves Its Mark on the Neural Substrates of Chinese Reading

**DOI:** 10.1523/ENEURO.0111-22.2022

**Published:** 2022-12-21

**Authors:** Jieyin Feng, Hoi Yan Mak, Jing Wang, Qing Cai

**Affiliations:** 1 Shanghai Key Laboratory of Brain Functional Genomics (Ministry of Education), Affiliated Mental Health Center (ECNU), Institute of Brain and Education Innovation, School of Psychology and Cognitive Science, East China Normal University, Shanghai 200062, China; 2 Department of Psychological Sciences, University of Connecticut, Storrs, CT 06269; 3 Department of Linguistics and Translation, City University of Hong Kong, Hong Kong 999077, China; 4 Shanghai Changning Mental Health Center, Shanghai 200335, China; 5 Shanghai Center for Brain Science and Brain-Inspired Technology, Shanghai 200062, China; 6 Haskins Laboratories, 300 George Street, New Haven, CT 06511

**Keywords:** Chinese, reading, reading acquisition, second language acquisition, writing

## Abstract

Understanding how the brain functions differently as one learns to read may shed light on the controversial nature of the reading ability of human being. Logographic writing system such as Chinese has been found to rely on specialized neural substrates beyond the reading network of alphabetic languages. The ability to read in Chinese has also been proposed to rely on writing skills. However, it was unclear whether the learning-related alteration of neural responses was language specific or resulted from the more reliance on writing practice during acquisition. This study investigated whether the emergence of typical logographic-specific regions relied on learning by writing. We taught proficient alphabetic language readers Chinese characters and used pre-test and post-test to identify changes in two critical stages of reading, namely, orthographic processing and orthographic-to-phonological mapping. Two typical left hemispheric areas for logographic reading showed increased responses to characters in the brains of proficient alphabetic readers after learning, regardless of whether the learning strategy involved writing practice. Moreover, learning strategy modulated the response magnitude or multivoxel patterns in the left superior parietal lobule, left middle frontal gyrus, and right fusiform gyrus, some of which were task dependent. The findings corroborated a limited role of writing in the emergence of logographic-specific reading network and suggested the heterogeneous nature of different brain regions in this network.

## Significance Statement

There has been debate on whether the development of skills for reading logographic characters depends on the skill of writing. We examined the emergence of typical Chinese-reading neural substrates when learners were taught character with and without training on writing. Behavioral and neural functional alterations were identified after proficient alphabetic readers learned to read Chinese with or without training on writing. Altering the responses in the left superior parietal lobule and middle frontal gyrus to Chinese characters did not require a writing-based learning, but writing modulated the responses in these areas.

## Introduction

Learning to read is one of the most remarkable capabilities of human being, the nature of which remains controversial. On the one hand, as a recent invention and a commonly mastered skill bound to language faculty, reading is proposed to be derived from preexisting functions and thus have universal neurobiological basis regardless of writing system (Paulesu et al., 2000; Dehaene et al., 2001, 2010; Cohen et al., 2002; Bolger et al., 2005; Dehaene and Cohen, 2007; Nakamura et al., 2012; Rueckl et al., 2015; Feng et al., 2020; Verhoeven and Perfetti, 2021). On the other hand, writing system is a product of culture and literacy acquisition is a product of education. Culture-specific views posit that reading is attuned to the characteristics of the specific languages (Tan et al., 2001, 2005a; Kuo et al., 2004; Siok et al., 2004, 2009; Hu et al., 2010).

Comparison between Chinese and alphabetic language reading has been a major arena for the debate. The writing system of Chinese is largely logographic: the orthography does not imply pronunciation. Chinese readers cannot rely on rules of orthographic-phonological mapping to decode the sound of a written word like one might do in alphabetic language reading. The critical role of phonological awareness in English reading development has been well documented (Wagner et al., 1997; Eden and Moats, 2002; Temple et al., 2003; Vellutino et al., 2004; Ziegler and Goswami, 2005; McArthur et al., 2018), whereas the reading development of Chinese as first or second language has been found to benefit much less from phonological skills (Meade, 2020) but more from the writing ability and orthographic awareness (Tan et al., 2005b; Ye et al., 2021; Wong and Zhou, 2022). Meta-analyses have consistently recognized the roles of several regions in orthographic processing and orthographic-phonological mapping during Chinese reading, including intraparietal sulcus/superior parietal lobule (IPS/SPL; Tan et al., 2005a; C.Y. Wu et al., 2012), left middle frontal gyrus (MFG; Bolger et al., 2005; Tan et al., 2005a; C.Y. Wu et al., 2012), and right ventral occipital-temporal areas (Bolger et al., 2005). Orthographic transparency affects the between-language similarity in brain activation patterns (Kim et al., 2016; Dong et al., 2021). For readers who are proficient at alphabetic language, successful learning of Chinese characters activates typical logographic processing areas (Y. Liu et al., 2007a; Nelson et al., 2009).

Regarding the original question on the nature of reading, the parallel cognitive and neural evidence of between-language difference does not address whether the neural activational differences result from the differences in the learning/processing strategy or the differences in the writing system itself. The covariant learning hypothesis proposes that the neural substrates for processing certain kind of stimulus are developed by associating the stimulus with the cognitive and learning processes (Kochunov et al., 2003; Tan et al., 2005a). The form of writing system affects the learning strategy, which affects the functional neuroanatomy of reading (Tan et al., 2005a). Thus, comparing how different learning strategies affect the processing of the same language is an effective approach to dissociating the effect of learning from that of the language per se. Several studies have investigated the effects of different learning strategies or cognitive processes on a given language (Y. Liu et al., 2007a; Nakamura et al., 2012; Cao et al., 2013, 2017; Cao and Perfetti, 2016; Lagarrigue et al., 2017). However, these studies have shown a mixed picture on whether the so-called logographic-specific areas are the results of training on writing, which might be because of the diversity in the training procedure, levels of processing, and the second language background of participants. Moreover, interpretation of the learning effect was also difficult in the absence of a prelearning functional neuroimaging measure when participants had no knowledge of the target language.

The present study investigated how the brain functions differently when proficient alphabetic language readers learned a novel logographic system in different strategies. We asked whether learning Chinese characters for a 7-d training elicited spontaneous neural responses in the typical Chinese reading areas and whether the emergence of these regions, particularly the SPL/IPS and MFG, relied on writing tutoring and practicing. Learning effect was examined using a pre-post test paradigm. We randomly assigned participants to two strategy groups and used a passive viewing task to examine the automatic processing of characters. We investigated two critical processes in reading acquisition at the very early stage, namely, character recognition (visual) and orthographic-phonological mapping (visual-auditory modality).

## Materials and Methods

### Screening of participants on language background and cognitive abilities

Language History Questionnaire (P. Li et al., 2014) was used to screen and recruit participants (1) whose age of first exposure to English was before 6, (2) whose self-evaluated proficiency of English was “very good” or “excellent,” (3) who were native speakers of Germanic or Romantic languages (Haspelmath et al., 2008; Lewis et al., 2014; Skirgård et al., 2017), and (4) whose experience with Chinese was minimal.

To ensure that participants were at very basic levels of Chinese, we presented a list that contained the 150 most-frequent Chinese characters (Cai and Brysbaert, 2010) and the real single-word characters used in the main experiment on a paper to each participant. Participants were asked to mark a character if they knew its pronunciation or meaning. Participants who marked over 10 characters were excluded from further study.

Handedness of participants was measured using a revised version of the Edinburgh Inventory (https://www.brainmapping.org/shared/Edinburgh.php, adapted from Oldfield, 1971). Only right-handed participants were included in further study.

The following tests were applied to ensure participants who were assigned to the two groups of learning strategies were of comparable language and cognitive abilities.

1. Multilingual Naming Test (MINT; Gollan et al., 2012). Participants were asked to name the black-and-white line-drawings in English one at a time. Accuracy was measured.
2. English vocabulary test. The test was originally developed for Dutch (Keuleers et al., 2015) and revised for testing English vocabulary (vocabulary.ugent.be/). The vocabulary set consisted of real words in American spelling and pseudowords. One word showed on the screen at a time and participants determined whether they knew this word or not by pressing the key “J” or “F.” The performance was indicated by *hit rate − false alarm rate*, i.e., the proportion of real words that were correctly recognized minus the proportion of pseudo-words that were mistakenly recognized as known words.
3. One Minute Reading Test (1MRT; Transvaal Education Department, 1987). Participants were asked to read across a page of English words out loud, from left to right, line by line, carefully but as fast as possible, for 1 min. All the words are one to two syllables.
4. Rapid Automatic Naming Test (RAN). The subtests of color-naming and digit-naming asked the participant to name the color or digit on the screen as fast as possible, and press any key to proceed to the next trial. Performance was measured by the reaction time on the correct trials.
5. Matrix span. The test was used to measure the visuospatial working memory (Stone and Towse, 2015). A five-by-five grid was shown on the screen in each trial. Some cells randomly changed color one by one. In the recall phase, participants were presented with the grid again and were asked to click on the cells in the order as they appeared. The number of cells to be remembered increased if the recall was correct. Memory span was the largest number of cells that could be correctly remembered.
6. Two-back verbal working memory test. In the English version, participants saw one word at a time and decided whether the stimulus in the *Nth* trial was the same as the one in the *N*–*2* trial. In the Chinese version of the task, the stimulus was individual Chinese characters. The ratio of “same” to “different” trials was 1:3. Performance was measured by accuracy.
7. Raven test. Participants took the short version of Raven test with 12 questions (Raven, 2017).

### Participants

Among the 81 adults from the ECNU and NYU-Shanghai community who volunteered to participate, 43 were excluded after screening: 35 for mismatched language background, seven for being left-handed, and one for early history of dyslexia. Among the 38 who participated in the study, three quit halfway, three fell asleep during fMRI scans, and the data of two were not fully recorded because of technical problems. This resulted in 30 participants in total (14 females) for the following analyses. All the participants were right-handed, age from 18 to 35, reported normal or correct-to-normal vision and normal hearing status, and had no history of neurologic disease or language impairment. Participants were native speakers of Germanic or Romantic languages and were proficient at English. All native English speakers rated their English proficiency as 7/7. For those whose native language was not English, the mean self-reported proficiency at English was 6.4/7 (very proficient or excellent), and the mean proficiency at their native language was 6.7/7. Fifteen participants were bilingual or multilingual, but none had experience with languages other than Germanic or Romantic languages. Their proficiency of Chinese was at very basic level (knew no more than 10 characters). They had started to learn the principles of using pinyin to code pronunciation of Chinese. This study was approved by the University Committee on Human Research Protection of East China Normal University (Approval Number: HR-0502017).

### Materials

The characters, pseudo-characters and scrambled characters or a subset of them were used in the learning session, behavioral pre-test and post-test, and the prelearning and postlearning fMRI tasks.

#### Real characters

One hundred and thirty-two Chinese characters were selected. These characters were of high frequency (Cai and Brysbaert, 2010; Table 1), each denoting to a concrete noun. There was no homophone in the stimuli. The characters were assigned to lists A and B, each containing 66 words. Character in the two lists were balanced on frequency (Cai and Brysbaert, 2010), frequency of their English equivalents (Brysbaert and New, 2009), age of acquisition, imageability (Y. Liu et al., 2007b), and stroke count (all *p*s* *>* *0.05; Table 1). The same radical never appeared in both lists.

**Table 1:** Mean and SDs of real characters

	Frequency of Chinese characters	Frequency of English words	Age of acquisition	Imageability	Stroke count
List A	3.58 (0.58)	3.22 (0.54)	3.48 (0.66)	6.31 (0.52)	8.29 (2.32)
List B	3.58 (0.54)	3.39 (0.61)	3.57 (0.61)	6.24 (0.66)	8.44 (2.27)
Mean (SD)	3.58 (0.56)	3.31 (0.58)	3.52 (0.64)	6.27 (0.59)	8.36 (2.29)

#### Pseudo-characters

One hundred and thirty-two pseudo-characters were produced based on the real characters used in this study. Radicals of the characters within a list were randomly shuffled and paired with the component of a different character using Truetype. We manually revised the stimulus if the generated one happened to be a real character.

#### Scrambled characters

Strokes of each pseudo-character was scrambled to create 132 scrambled characters using Truetype. We purposely adjust some scrambled characters to ensure that the structure did not follow orthography principles of Chinese.

#### Evaluations of pseudo-characters and scrambled characters

An independent group of 23 English native speakers with Chinese-learning experience over one year were recruited to assess the character-ness of pseudo-characters and scrambled characters. The raters saw the stimuli in a randomized order, one at a time, and rated “To what extent do you think this is a Chinese character” on a five-point scale. The mean resemblance score of pseudo-characters was 4.05 (SD = 0.51) and the mean of scrambled characters was 1.04 (SD = 0.08). The mean rating of the pseudo-characters was significantly greater than that of the scrambled characters (one-tailed *t* = 67.27, *p* < 0.00001), suggesting good validity of the stimuli.

#### Auditory stimuli

Characters were read by a Mandarin Chinese native speaker. The recorded audios were equated on loudness, frequency band and bit rate using Adobe Audition. One hundred and thirty-two nonverbal sound was created by reversing the audio of each character. Audios of tones at 500, 600, and 700 Hz were created.

### Design and overall procedure

**Figure 1.**
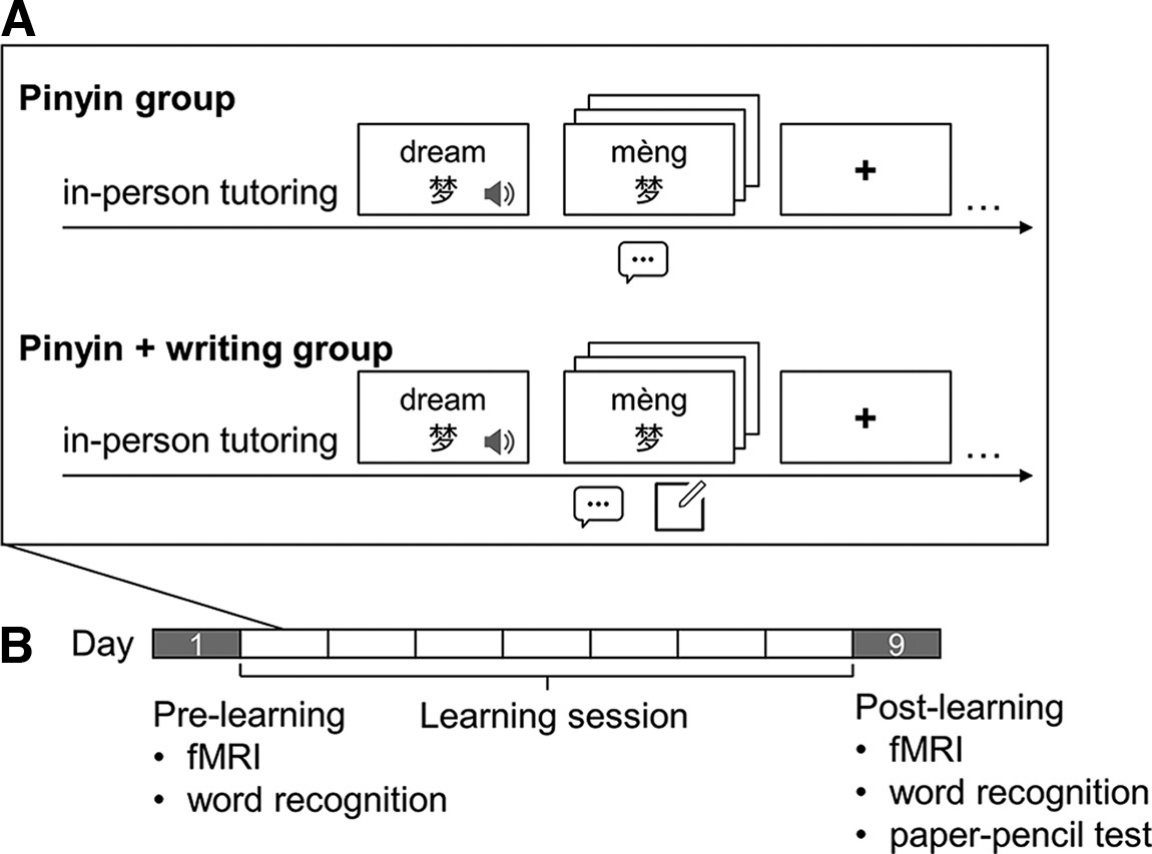
***A***, Paradigms of character learning in two strategy groups. ***B***, Timeline of the study for each participant.

Participants were assigned to one of the two groups of learning strategy, fifteen in each group, after the screening tests. Each participant went through the prelearning behavioral test and fMRI scan on day 1 and went through the postlearning test and scan on day 9 (Fig. 1). On days 2–8, they learned 66 Chinese characters by either a pinyin-based strategy or a pinyin + writing strategy according to the group assignment. According to the screening tests, no significant between-group difference was found in the performance of any cognitive ability test, English vocabulary, MINT, or RAN-digit test (all *p*s* *>* *0.05). The pinyin group showed higher accuracy in the 1MRT English reading test (*t* = 2.46, *p* = 0.02) and shorter reaction time in the RAN-color naming test (*t* = −2.11, *p* = 0.04), likely because there were 11 English monolinguals in the pinyin group and only 4 in the other.

### Learning session

Each participant studied all the 66 real characters in one of the real-character lists (Fig. 1). In each strategy group, half of the participants learned list A and the other half learned list B. The learning session lasted for seven consecutive days, including five acquisition phases and two review phases. The first review phase was on day 4 and the second was on day 8. In each acquisition phase, participant received a list of 13 or 14 new characters. Experimenter first went over the pinyin of each character with participants to ensure that they were able to pronounce the characters using pinyin. For the pinyin + writing group, experimenter also taught participants the basics of character writing, including identifying the subcomponent of a character, writing individual strokes, and writing with correct stroke order. Then the participant went over the characters on a program implemented in E-prime 2.0. A character and its English translation were shown on the screen for 1 s while the pronunciation was played once. The next slide presented the character and its pinyin. The pinyin group were asked to spell the pinyin and pronounce the character. The pinyin + writing group were asked to additionally write down the character. The practice slide was learner-paced and repeated for three times. At the end of an acquisition phase and the beginning of a new acquisition phase, participants took a spelling test, during which they wrote down the pinyin of a heard character that was learned in this/the previous phase. The pinyin + group was asked to additionally wrote down the character.

In the two review phases, participants took the spelling and dictation test of same paradigm as those at the end and beginning of an acquisition phase, except that the target characters in the review-phase tests included all the words that had been learned.

### Prelearning and postlearning behavioral tests

#### Character recognition paradigm

On day 1 and day 9, participants performed character recognition tasks in visual, auditory, or visual-auditory modality. In the visual task, participants judged whether they knew the character on the screen. For each participant, materials were the 66 learned real characters and 66 derivative pseudo-characters. In the auditory task, participants judged whether the pronunciation refers to a Chinese word. Stimuli were the pronunciation of learned characters for each participant and the corresponding reversed audios. The auditory task was irrelevant to the aim of the present study and was not considered in further analyses. In the visual-auditory task, participant judged whether the character on the screen matched the auditorily presented speech sound. Stimuli were the 66 learned characters, each paired with either the correct pronunciation or the pronunciation of another character. In each trial, the target stimulus was presented for 600 ms, followed by a 2000-ms blank screen, during which participant responded by pressing buttons. Accuracy was recorded. Trials within each modality were randomized and separated into two blocks with equal number of trials. The presentation order of the blocks was randomized. Participants could take break between blocks.

#### Behavioral data analysis

Two-way mixed-design ANOVA was performed to examine the effect of learning on accuracy of character recognition test and the effect of strategy on the learning effect. The within-subject factor was the stage of learning (prelearning vs postlearning) and the between-subject factor was the learning strategy (pinyin + writing vs pinyin). The tests were performed on different stimulus modalities separately. Note that we only examined the recognition rate of real characters, because the decision on pseudo-character did not reflect a learning effect.

### Prelearning and postlearning task in fMRI

**Figure 2.**
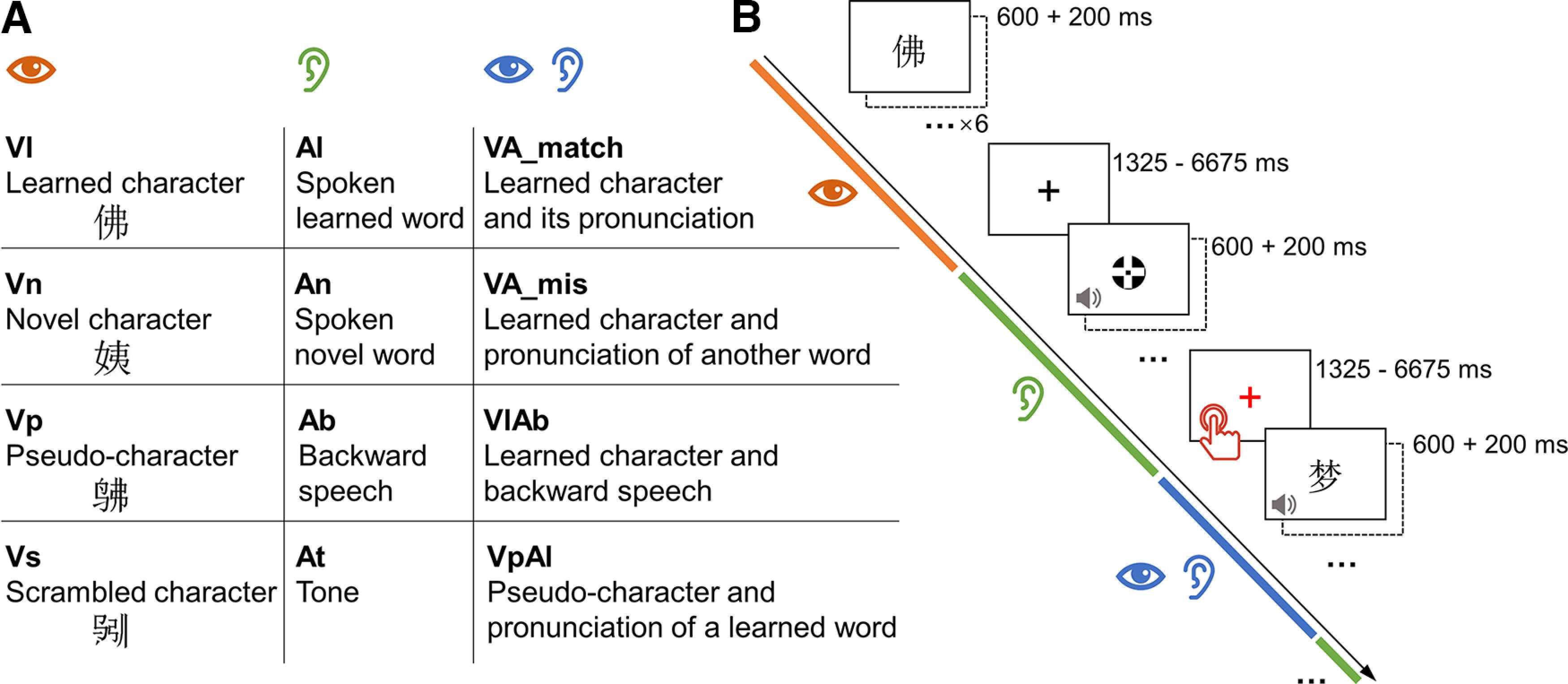
Conditions (***A***) and paradigm (***B***) of the fMRI language task. Sequence of the visual (orange), auditory (green), or integrated (blue) miniblocks were randomized. Participants were asked to press the button when a fixation was shown in red.

The fMRI task used a block design. Participants read or listened to the learned characters and other stimulus (see above, Materials*)* and performed a perceptual detection task while being scanned. Because each participant learned only half of the 132 words, characters in the unlearned list were used as the novel characters for the participant. The main experiment implemented a mixed design. The learning strategy (pinyin + writing vs pinyin) was a between-subject variable. The stage of learning (prelearning vs postlearning) and the type of stimuli were the within-subject variables. There were twelve types of stimulus (Fig. 2*A*): visually presented learned character (Vl), novel character (Vn), pseudo-character (Vp), and scrambled character (Vs); auditorily presented pronunciation of learned character (Al), novel character (An), reversed speech sound of learned word (Ab), and tone (At); learned character and its pronunciation (VA_match), learned character and pronunciation of another word (VA_mis), learned character and the reversed speech sound (VlAb), and pseudo-character and pronunciation of learned word (VpAl). A stimulus trial was formed of a 600 ms stimulus and a 200 ms blank (Fig. 2*B*). The presentation sequence of the 66 stimulus trials in each condition were pseudo-randomized and grouped into 11 miniblocks, six trials per block. Sequences of the miniblocks of all the conditions were determined using OPTSEQ (https://surfer.nmr.mgh.harvard.edu/optseq/). A fixation cross was presented between miniblocks, the duration of which was jittered, ranging from 1.625 to 6.675 s. Twenty-one out of the 66 fixations were presented in red and randomly distributed through the task. Participants were asked to passively view and listen to the stimulus, and press a button as soon as a red fixation appeared. The task was separated into two runs and took around 20 min in total.

### MRI acquisition

Subjects were scanned in a 3T MRI scanner (Siemens Prisma; Siemens) using a 20-channel head coil. Functional images were acquired using a single-shot T2*-weighted gradient-echo echo planar imaging pulse sequence [TR = 2450 ms, TE = 30 ms, flip angle (FA) = 81°, each volume comprising 40 slices, matrix 64 × 64, field of view (FoV) = 192 mm × 192 mm^2^, voxel size = 3 × 3 × 3 mm^3^, interleaved acquisition]. T1-weighted anatomic image was acquired using a multiecho MPRAGE sequence (TR = 2300 ms, TE = 2.32 ms, FA = 8°, matrix 256 × 256, FoV = 240 × 240 mm^2^, slice thickness = 0.9 mm).

### Image preprocessing

Image preprocessing and uni-voxel analyses were performed using SPM8 (Wellcome Centre for Human Neuroimaging, London; https://www.fil.ion.ucl.ac.uk/spm/). The first four volumes of each session were excluded to allow for magnetic saturation. Functional images were corrected for slice timing and head motion, normalized to MNI space using the segmentation-based procedure, smoothed using a Gaussian filter [full-width at half-maximum (FWHM) = 5 mm], and filtered with a 128-s high-pass filter. The moderate kernel size was applied so that the local multivoxel patterns were retained.

### Whole-brain uni-voxel analysis

The main effect of learning and the effect of strategy on the learning effect were examined in the two-stage random-effect analyses using general linear model (GLM). Subject-specific responses to each of the 12 types of stimuli were estimated in the prelearning and postlearning scans separately using general linear models, regressors of which were constructed as a boxcar function convolved with the canonical hemodynamic response function. Trials of participant responses and the six rigid-body motion parameters were modeled as covariates.

To examine the effect of learning, the within-subject effect of the post > prelearning contrast was first estimated in the first-level GLM for each participant. Second-level analysis was performed on the contrast images over all participants using one-sample *t* test against zero. To examine the interaction between strategy and learning stage, specifically, the effect of strategy on the effect of learning, the first-level contrasts of post > prelearning were supplied to the second-level between-group test, where the pinyin versus pinyin + writing groups were compared using independent sample *t* contrast. Each group-level contrast map was thresholded at a cluster-wise corrected α of 0.05 using the AlphaSim algorithm implemented in NeuroElf (https://neuroelf.net/). Significance of the clusters was determined jointly by the voxel-wise *p* of 0.05 and the minimum cluster size determined by a 2000-iteration simulation.

### Uni-voxel region of interest (ROI) analysis

We performed ROI analyses to further investigate the effect of strategy on learning, specifically, whether involving a writing-based learning strategy will lead to response differences in the regions that have been consistently identified to be specific to Chinese reading. Four coordinate-based a priori regions of interests (ROIs) were selected based on previous meta-analyses on Chinese character reading: left SPL, left MFG, and the right and left FG. The left SPL, left MFG and right FG were considered Chinese-specific and reliably identified in multiple subcomponents of character processing (see Introduction). Because the fusiform ROI was the only ROI in right hemisphere and because it was part of the language-general reading network, we additionally included the left FG as a left hemispheric benchmark to the right ROI. Each ROI was constructed as a 12-mm radius sphere centered at the peak coordinate reported by the meta-analyses. The coordinates of the left MFG ([−46, 18, 28]) and SPL ([−36, −42, 48]) were from Tan et al. (2005a), because this was the meta-analysis that revealed these areas by directly contrasting the Chinese reading against alphabetic reading. The coordinate of the right FG (converted to MNI coordinate from Talairach coordinate [33, −67, −14]) was from Bolger et al. (2005), because this was the meta-analysis that identified the right FG in Chinese reading, and this was the review that proposed the right occipitotemporal cortex was more involved in Chinese reading. While the left FG was a universally identified area for reading, we used the coordinate identified for Chinese character processing ([−32, −54, 6]) in the meta-analysis by Tan et al. (2005a). The first-level contrast of post > prelearning was averaged across voxels within each ROI within participant. The mean signals were compared between two strategy groups over participants using independent sample t contrast. Because reading development of Chinese has been found to behaviorally benefit from writing ability (Tan et al., 2005b; Ye et al., 2021; Wong and Zhou, 2022; see Introduction), and because the selected ROIs have been suggested specific to Chinese reading, we examined whether the additional writing training resulted in increased activations in these regions, by performing the pinyin + writing > pinyin group contrasts.

### Multivoxel pattern analysis in a priori ROIs

We performed classification analyses to examine whether the learning strategy affected the multivoxel patterns associated with character processing in each of the four ROIs. Training and testing were performed in a cross-validation procedure that iterated over participants. In each cross-validation fold, all but one participant’s data were used for training and the left-out participant’s data were used for testing. The training exemplars were the voxel patterns of the post > prelearning contrast within each ROI of the training participants. The training label/target was the group membership of each participant, i.e., the learning strategy (pinyin or pinyin + writing). Support vector machine classifiers were trained to learn the neural signatures associated with each of the two strategies. The trained classifiers were applied to predict the group membership of the left-out participant. The mean accuracy over all folds, i.e., participants, indicated whether the neural signatures of learning effect were systematically altered by the learning strategy so that they could be used to predict the learning strategy used by an individual whose data were previously unseen by the model. The significance level of the accuracy was determined by 2000-iteration random permutations, in which all the procedure and data remained the same as the actual analyses, except that the labels of the test exemplars were randomly shuffled within each fold.

### Language background classification

The different number of monolingual and bilingual participants in the two groups was a potential confounder to the effect of strategy. Therefore, we performed classification on the language background of participants using exactly the same data of interests as in the main analysis, namely, the a priori ROIs and the clusters identified by the whole-brain analysis on strategy effect. The same procedure of multivoxel pattern analysis of strategy classification was applied, except that the training label/target was the language background of a participant, i.e., being monolingual versus bilingual. If the classification accuracy was not significantly different from the chance level, it suggested that the neural signatures associated with the tasks between monolingual and bilingual participants were not distinguishable, hence, the group difference could not be attributed to the language background. The data and scripts will be shared on request.

## Results

### Behavioral results on character recognition test

**Figure 3.**
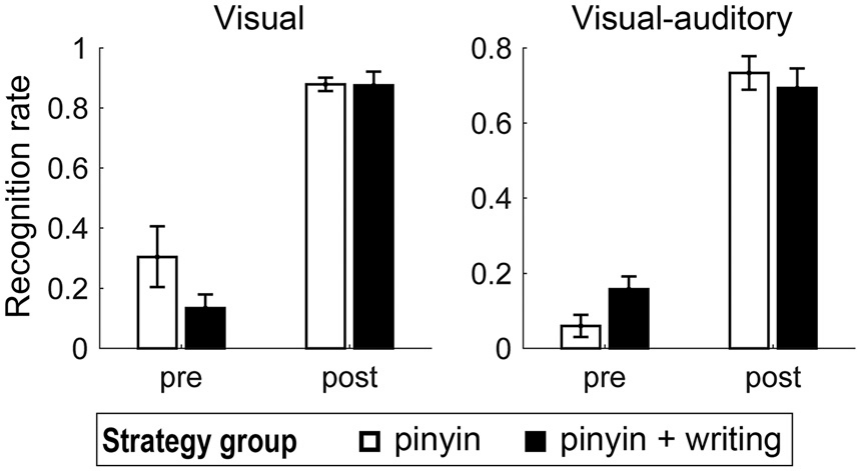
Behavioral results of character recognition rate.

In the visual modality of the character recognition test, ANOVA revealed a main effect of the stage of learning (*F*_(1,28)_ = 125.55, *p* = 6.14 × 10^−12^) on recognition rate. Learning improved the performance from a mean recognition rate of 0.21–0.88 (Fig. 3). No significant effect of learning stage × strategy interaction (*F*_(1,28)_ = 12.06, *p* > 0.05) or main effect of strategy (*F*_(1,28)_ = 1.91, *p* > 0.05) was found. In the visual-auditory test, ANOVA also revealed a main effect of the stage of learning (*F*_(1,28)_ = 277.09, *p* = 4.70 × 10^−16^) on the performance. Learning improved the recognition rate from a mean of 0.10–0.71 (Fig. 3). No significant effect of learning stage × strategy interaction (*F*_(1,28)_ = 3.63, *p* > 0.05) or main effect of strategy (*F*_(1,28)_ = 0.39, *p* > 0.05) was found. Thus, different learning strategies did not cause different learning gains in the recognition test. The postlearning recognition rate over all participants in both tasks ranged from 0.20 to 0.98, suggesting that the performance did not reach a theoretical ceiling.

### Effects of learning: whole-brain uni-voxel GLM results

#### Effect of learning on orthographic processing

**Figure 4.**
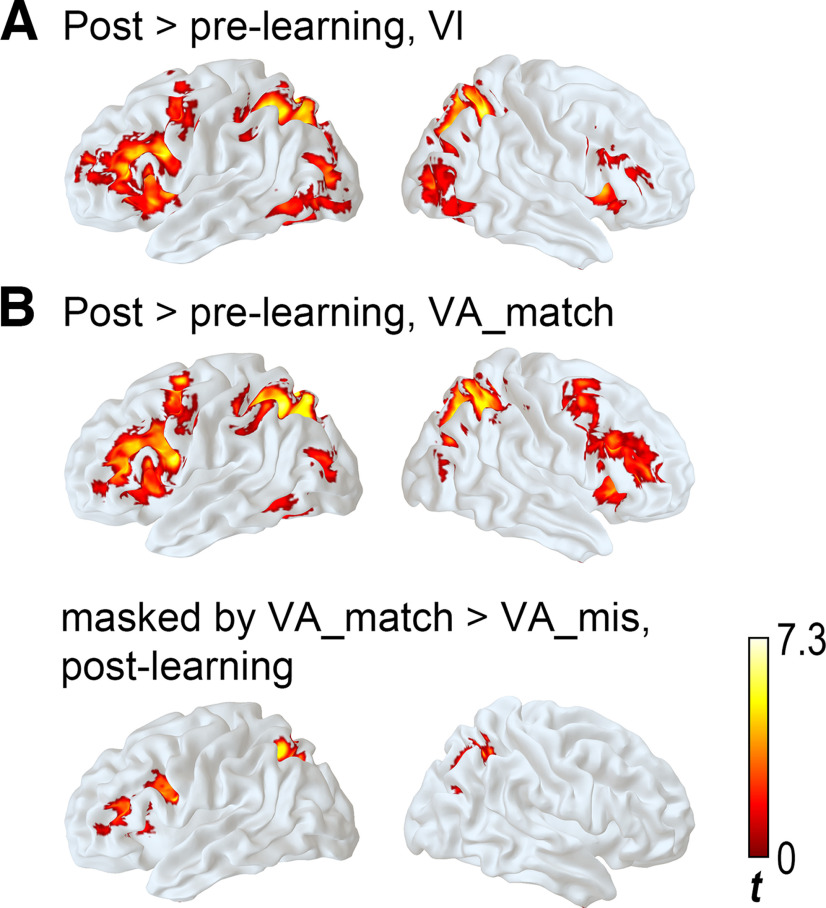
Whole-brain uni-voxel results of learning effect. ***A***, Results of the visual processing. ***B***, Results of visual-auditory processing.

For visually presented characters that were studied during the learning session (*Vl*), the main effect of learning (postlearning > prelearning contrast over both strategy groups) was found in wide cortical areas in the bilateral SPL that extended to middle occipital gyrus (MOG), the left inferior gyrus (IFG) that included pars opercularis and pars triangularis and extended to MFG, the supplementary motor areas (SMA), and the right insula-IFG (Fig. 4*A*; Table 2; cluster-wise corrected *p *=* *0.05, cluster size determined by voxel-wise *p* of 0.05; df* *=* *29). By contrast, activational difference between postlearning and prelearning scans for the scrambled characters were observed in one cluster at the calcarine-precuneus area (peak coordinate *x* = 0, *y* = −61, *z* = 19; peak Z = 4.49, K = 314 voxels; cluster-wise corrected *p* = 0.05, cluster size determined by voxel-wise *p* of 0.05; df* *=* *29).

**Table 2:** Regions presenting the main effect of learning

Postlearning > prelearning	H	BA approx.	K (voxels)	MNI coordinate			Z
				*x*	*y*	*z*	
Visual							
Supplementary motor area	-	6	731	0	20	49	5.03
Paracingulate gyrus		24		−6	26	31	3.94
Anterior cingulate cortex		24		6	26	28	3.66
Middle occipital gyrus	R	19	553	30	−73	34	4.91
Superior parietal lobule		7		30	−61	55	4.78
Superior parietal lobule		7		30	−55	49	4.74
Superior parietal lobule	L	7	760	−27	−64	52	4.73
Inferior parietal lobule		7		−33	−49	49	4.67
Middle occipital gyrus		19		−24	−67	34	4.44
Inferior frontal gyrus	L	44	1520	−42	8	22	4.60
Insula		48		−42	8	4	4.21
Inferior frontal gyrus		45		−48	29	13	4.07
Insula	R	48	312	33	14	1	3.91
Inferior frontal gyrus		44		57	17	28	3.51
Inferior frontal gyrus		45		45	17	22	3.29
Visual-auditory							
Inferior frontal gyrus	L	44	1575	−48	8	16	4.99
Middle frontal gyrus		9		−30	−1	61	4.20
Middle frontal gyrus		9		−42	2	55	4.15
Inferior parietal lobule	L	40	2284	−30	−46	40	4.93
Superior parietal lobule		7		−27	−64	55	4.82
Superior occipital gyrus	R	7		27	−64	43	4.78
Inferior frontal gyrus	R	44	947	54	17	25	4.25
Inferior frontal gyrus		45		48	38	10	4.02
Insula		48		30	23	10	3.84
Supplementary motor area	R	6	392	0	17	55	4.09
Paracingulate gyrus		32		12	26	40	3.81
Supplementary motor area		8		6	23	49	3.81
Inferior temporal gyrus	L	37	166	−48	−52	−11	3.61
Fusiform gyrus		37		−39	−58	−8	3.50

H: hemisphere; L: left; R: right; BA approx.: approximated Brodmann area. Regions with indented names were subclusters. The note also applies to Table 3.

#### Effect of learning on visual-auditory processing

When the character and its pronunciation were presented simultaneously (*VA_match*), the post > prelearning effects across strategy groups were found in the left IFG extending to MFG, the bilateral SPL-superior occipital gyri, the left inferior temporal gyrus-FG, the right IFG, and the SMA (Fig. 4*B*; Table 2; cluster-wise corrected *p* = 0.05, cluster size determined by voxel-wise *p* of 0.05; df* *=* *29).

To examine whether the identified areas were sensitive to the correct orthographic-phonological mapping, or whether they just reflected a general effect of multimodal processing, we performed the *VA_match versus VA_mis* contrast in the postlearning session, thresholded the map using the same cluster-wise corrected p at 0.05, and masked the results of the learning effect with the *VA_match versus VA_mis* contrast results. Significant effects were identified in the left IFG-MFG and bilateral SPL-MOG (Fig. 4*B*), suggesting that these areas were sensitive to the correct speech-print association. That is, among the regions showing the main effect of learning, the SMA, left inferior temporal gyrus, and right IFG were not found to respond differently to matched versus mismatched speech-print pairing.

### Effects of strategy: whole-brain uni-voxel GLM results

#### Effect of strategy on orthographic processing

**Figure 5.**
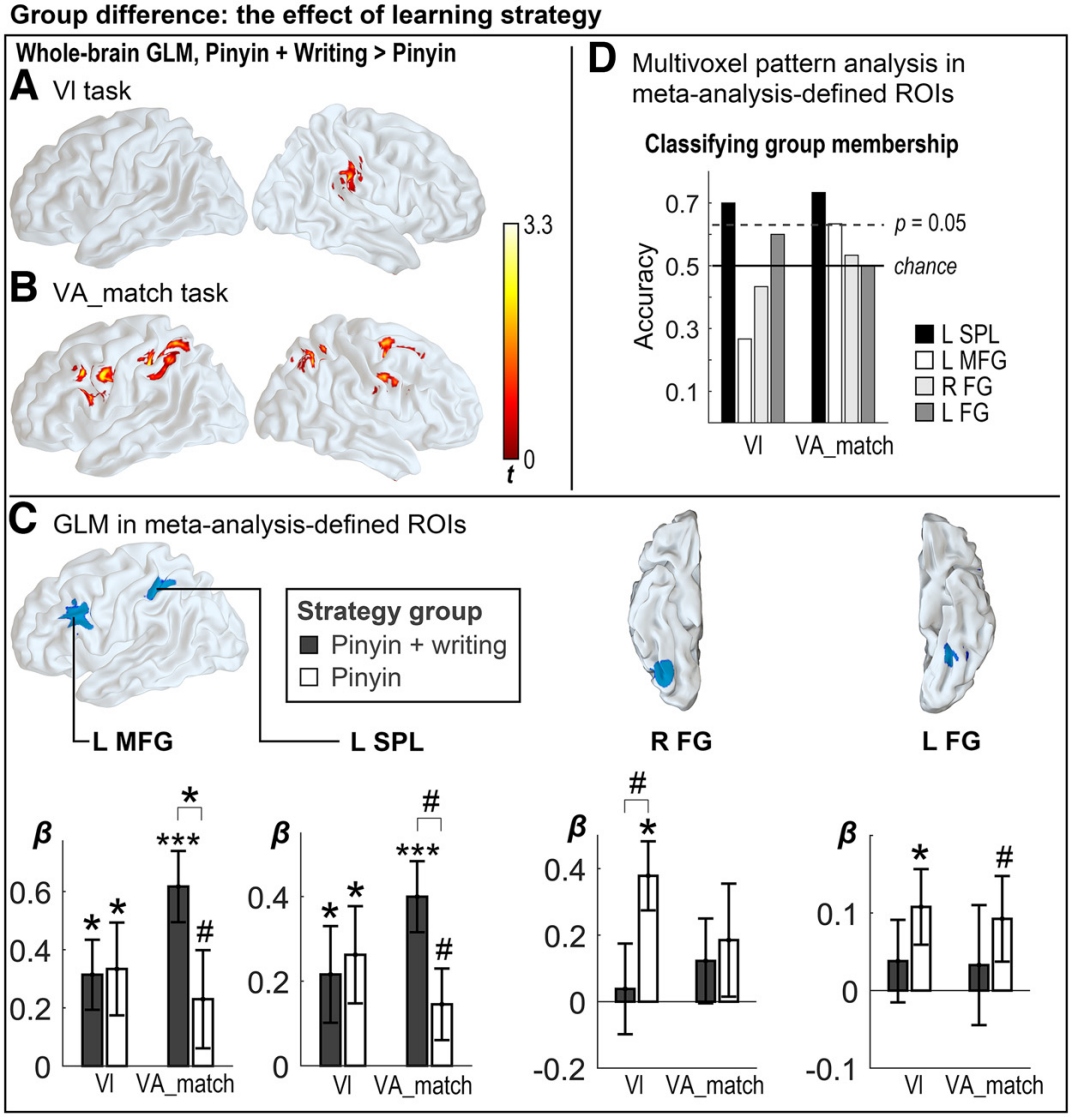
Effect of strategy on learning effect. ***A***, Whole-brain uni-voxel GLM results for Vl processing. ***B***, Whole-brain uni-voxel GLM results for the VA_match processing. *Post hoc* analysis of each cluster was shown in Extended Data Figure 5-1. ***C***, ROI uni-voxel analysis results. The four ROIs defined based on meta-analyses were shown in surface rendering of the brain. Mean and SE of the β estimates for each ROI in each condition were shown in the bar graphs. Markers above a bar indicated the mean was significantly greater than zero according to a one-sample *t* test. Markers between two bars indicated significant mean difference between groups. ***p < 0.001, *p < 0.05, #*p* < 0.1. The overlap of the a priori ROIs and results of the whole-brain analysis was shown in Extended Data Figure 5-2. ***D***, Accuracy of classifying participant’s group membership. Note that the critical values of accuracy at *p* of 0.05 were determined based on random permutation of each set of data independently, but all the critical values turned out to be the same (displayed as a dashed straight line), which was not surprising when the numbers of cross-validation folds and the numbers of test exemplars per fold were the same across all the classification tests. The results of classifying participant’s language background using the same procedure were as shown in Extended Data Figure 5-3.

The between-group contrast revealed that in *Vl* processing, the learning effect for the pinyin + writing group was greater than that for the pinyin group in two adjacent clusters in the right supramarginal gyrus (SMG) and postcentral gyrus (Fig. 5*A*; Table 3; cluster-wise corrected *p* = 0.05, cluster size determined by voxel-wise *p* of 0.01; df* *=* *28). *Post hoc* analysis revealed that the effects in both clusters were contributed by a decrease in activation after learning (postlearning < prelearning) of the pinyin group (Extended Data Fig. 5-1).

**Table 3:** Regions presenting difference between strategy groups (pinyin + writing > pinyin) on the learning effect

Pinyin + writing > pinyin	H	BA approx.	K (voxels)	MNI coordinate			Z
				*x*	*y*	*z*	
Visual							
Postcentral gyrus	R	3	98	39	−19	31	3.15
Supramarginal gyrus		40		36	−31	31	2.82
Supramarginal gyrus		40		45	−34	31	2.49
Supramarginal gyrus	R	40	50	60	−28	46	2.46
Supramarginal gyrus				63	−28	34	2.33
Supramarginal gyrus				66	−31	25	2.26
Visual-auditory							
Precentral gyrus	L	4	152	−48	−4	28	3.00
Middle frontal gyrus		9		−51	8	37	2.97
Middle frontal gyrus	R	9	84	24	11	49	2.92
Superior frontal gyrus		8		21	5	43	2.23
Precentral gyrus		6		27	−16	49	2.21
Intraparietal sulcus	L	40	135	−42	−37	40	2.78
Superior parietal lobule		7		−42	−52	58	2.28
Supramarginal gyrus		40		−51	−37	37	2.26
Angular gyrus	R	39	57	39	−67	49	2.73
Superior parietal lobule		7		42	−55	55	2.09
Precentral gyrus	R	4	51	51	−4	37	2.49
Precentral gyrus		6		54	5	31	2.23
Postcentral gyrus		43		54	−10	28	1.74

10.1523/ENEURO.0111-22.2022.f5-2 Extended Data Figure 5-2 Overlap of a priori ROIs (in blue) and results of the whole-brain analysis of strategy effect (pinyin + writing > pinyin; in red). The overlapped areas are shown in pale pink. Download Figure 5-2, TIF file.

10.1523/ENEURO.0111-22.2022.f5-3 Extended Data Figure 5-3 Accuracy of classifying participant’s language background. Download Figure 5-3, DOC file.

10.1523/ENEURO.0111-22.2022.f5-1 Extended Data Figure 5-1 *Post hoc* analysis of the effect of strategy on the learning effect. Mean and SE of each cluster in the two tasks for the pinyin (P) and pinyin + writing (P + W) groups was plotted. Download Figure 5-1, TIF file.

#### Effect of strategy on visual-auditory processing

The between-group contrast revealed that in *VA_match*, learning effect for the pinyin + writing group was greater than the pinyin group in the left precentral gyrus (PrC) that extended to the MFG, the left IPS/SPL, the right MFG-PrC, and right angular gyrus (cluster-wise corrected *p* = 0.05, cluster size determined by voxel-wise *p* of 0.05; df* *=* *28; Table 3; Fig. 5*B*). *Post hoc* analysis revealed that for all but one cluster, the pinyin + writing group showed an increase in activation after learning, whereas the pinyin group did not show substantial learning effect in these areas. The only exception was the cluster centered at the right PrC, in which the pinyin + writing group had a marginally significant positive effect, and the pinyin group showed a significant decrease in activation (Extended Data Fig. 5-1).

### Effects of strategy: uni-voxel ROI analysis

The effect of learning strategy was further investigated in coordinate-based predefined ROIs. The clusters revealed by the whole-brain analyses spatially overlapped with the predefined ROIs of left SPL and MFG (Extended Data Fig. 5-2). In the VA_match task, the left SPL presented greater activations for the pinyin + writing than the pinyin group (*t* = 2.06, two-tailed *p* = 0.048), and a marginal effect was found in the left MFG (*t* = 1.79, two-tailed *p* = 0.084; Fig. 5*C*). By contrast, no ROI showed group difference when the presented word form and pronunciation were mismatched.

In the Vl task, no ROI showed greater learning effect for the pinyin + writing group than the pinyin group. However, a tendency of greater learning effect for the pinyin group compared with the pinyin + writing group was found in the right FG (pinyin versus pinyin + writing, *t* = 1.92, two-tailed *p* = 0.066; Fig. 5*C*). Similar patterns were observed during the scrambled word processing (Vs) in the bilateral fusiform gyri: The pinyin + writing group showed a decreased activation after learning, and learning resulted in greater responses for the pinyin group than the pinyin + writing group (post > prelearning in the versus processing, right FG: M_p+w_ = −0.33, M_p_ = 0.10, SD_p+w_ = 0.51, SD_p_ = 0.28, pinyin versus pinyin + writing, *t* = 2.73, two-tailed *p* = 0.011; left FG: M_p+w_ = −0.14, M_p_ = 0.02, SD_p+w_ = 0.22, SD_p_ = 0.12, pinyin versus pinyin + writing, *t* = 2.27, two-tailed *p* = 0.031). The SPL or MFG did not reveal a group difference during the versus processing.

### Effects of strategy: multivoxel pattern analysis within ROI

The group membership of each participant, namely, the pinyin or the pinyin + writing group, was identified based on the multivoxel patterns of other participants. The classifications resulted in mean accuracies of 0.73 and 0.70 when using the multivoxel patterns within left SPL during the VA_match and Vl processing respectively, both of which were significantly higher than the chance-level accuracy of 0.5 (Fig. 5*D*; random permutation-based significance tests, *p* < 0.05). In the left MFG, only the multivoxel patterns during VA_match processing showed a marginally significant mean accuracy of 0.63 (*p* = 0.05). Patterns of these two regions during the scrambled character processing or mismatched sound-print processing were not distinguishable between the groups of participants (all *p*s* *>* *0.05): the accuracies were 0.50 (VA_mis in SPL), 0.60 (vs in SPL), 0.23 (VA_mis in MFG), and 0.3 (vs in MFG), respectively.

Classification accuracy within the right or left FG was not significantly different from chance level (Fig. 5*D*). However, during scrambled character processing, the patterns in the right FG resulted in a marginally significant accuracy of group identification at 0.63 (*p* = 0.05).

### Results of language background classifications

Based on the multivoxel patterns in either ROI during either Vl or VA_match processing, the accuracies of classifying individual participants as being monolingual or bilingual were not significantly above chance level, ranging from 0.33 to 0.57 (Extended Data Fig. 5-3). Based on the multivoxel patterns in the clusters that presented the effect of strategy in the whole-brain analysis, the mean accuracy of classifying language background over participants was 0.53 (SD = 0.50) for the Vl processing and was 0.47 (SD = 0.50) for the VA_match processing, neither being significantly above chance level. These results suggested the effect of strategy group identified in the previous analyses was not an effect of bilingualism.

## Discussion

This study investigated the neural functional alterations associated with learning to read Chinese as a second language. By using the pre-test and post-test paradigm, we observed spontaneous changes in two critical stages of reading, namely, the orthographic processing and the orthographic-to-phonological mapping. Typical areas for logographic reading in the brains of proficient alphabetic readers became more responsive in superficial processing of Chinese inputs after a week of learning. Although the behavioral learning effect was not strategy dependent, whether or not involving the writing practice modulated the neural responses of the left superior parietal cortices, left middle frontal gyrus, and the right fusiform gyrus, and these modulations were observed in different tasks associated with character processing.

Learning altered the neural responses in some of the areas that have been found to be commonly activated in reading in different languages, including the left IFG, left insula, the SMA and the adjacent anterior cingulate cortex, the left fusiform gyrus, and the bilateral extrastriate cortices (Tan et al., 2005a; Maisog et al., 2008; Richlan et al., 2009; Rueckl et al., 2015). Among these regions, the IFG-insula has been identified as one of the language-general speech-print convergence regions, in that this area showed correlated responses to visual and auditorily presented words in multiple languages, including Chinese (Rueckl et al., 2015). Moreover, the logographic-specific areas in the left MFG and left SPL also showed increased activation after learning. Activation of the typical alphabetic reading-related areas in the left posterior temporal gyrus and left angular gyrus were not found altered by reading Chinese. The above-mentioned results have been observed during both the visual and the visual-auditory processing. These findings were consistent with the view that reading Chinese as a second language showed an accommodation pattern (Nelson et al., 2009; Ma et al., 2020). The present results further revealed that the accommodation appeared in broader reading networks in addition to the visual perceptual processing areas.

Although the postlearning improvement of behavioral performance was not affected by learning strategy, the interaction of strategy and learning stage in neural response suggested some logographic-specific areas were modulated by whether the learning involved writing practice. Some previous behavioral studies have shown the effect of handwriting practice on literacy acquisition (Wiley and Rapp, 2021) or the correlation between the two abilities (Tan et al., 2005b), while others have found a dissociation between writing and reading in Chinese. For instance, knowledge of how a character was written did not influence character processing in proficient readers (Zhai and Fischer-Baum, 2019). Patient with left temporoparietal lesions presented complete writing deficits and poor orthographic awareness, but was able to perform perfectly in Chinese reading task (Bi et al., 2009). The present results showed the effect of writing on reading was limited from a neural perspective: For the passive viewing process, uni-voxel analyses revealed that learning by pinyin decreased the activations in the right supramarginal gyrus, which contributed to the group difference in the learning effect (Fig. 5*A*; Extended Data Fig. 5-1), whereas the multivoxel analysis showed that writing also altered the activation patterns of the left SPL. Although the right SMG is not considered part of the canonical reading network, it has been identified as a cross-language speech-print convergence area, where the response magnitudes to written and spoken words are correlated over participants (Rueckl et al., 2015). An inference according to this view is that learning characters by associating the visual form with pinyin has resulted in less reliance on this universal sound-print association area, and potentially more reliance on the Chinese-specific neural substrates, such as the left MFG and SPL as identified in the visual-auditory processing.

During visual-auditory processing, the effects of learning strategy were driven by the greater postlearning increase of the pinyin + writing group compared with the pinyin group. The identified brain areas can thus be viewed as areas to which the additional writing practice has brought additional response increase. These results have shown spatial overlap with the meta-analysis-determined logographic-specific ROIs (Extended Data Fig. 5-1) in the left SPL and MFG. The joint results of uni-voxel and multivoxel analyses on strategy effect have suggested that (1) both strategies have increased the sensitivity of left SPL to characters; (2) the responses of L SPL to characters are modulated by learning strategy; (3) involving writing practice during learning tends to slightly increase the MFG response only during the orthographic-phonological mapping; and (4) the right FG is modulated by strategy during the uni-modal visual processing, but this effect is not orthographic-specific, which differ from the response of the left FG. We discuss these implications by ROIs below.

### Left superior parietal lobule

First, learning led to greater responses of the left SPL in both the orthographic (Vl) and the orthographic-phonological (VA_match) processing for both of the strategy groups. Second, adding writing-based learning caused greater responses during the orthographic-phonological processing as compared with the pinyin-only strategy. Third, while the uni-voxel analyses revealed no group difference in the response magnitude during the orthographic processing, the between-group differences in multivoxel patterns associated with the orthographic processing were reliable enough to predict the learning strategy used by individual learners according to the multivoxel patterns of other learners. Forth, this area was unable to identify participant’s learning strategy when the displayed character was paired with a wrong pronunciation, suggesting the strategy effect was sensitive to the congruency of multimodal input, or in other words, the strategy effect was manifested based on participants’ knowledge about character identity. Therefore, the activities in left SPL during both orthographic processing and orthographic-phonological mapping tasks relied on how the characters were learned. The findings concur with the proposal for IPS/SPL being part of the reading network that is specialized for fine-grained visuospatial analysis and motor gesture inference (Kuo et al., 2004; Siok et al., 2009; Nakamura et al., 2012). The IPS/SPL has been found to be sensitive to visual distortion of word (Nakamura et al., 2012), activate less in a size judgment task (Siok et al., 2009), and show decreased resting-state functional connectivity with the left MFG (Zhou et al., 2015) in Chinese dyslexic children compared with the nondyslexics, suggesting the role of SPL in normal reading might not be just correlational. Our results further suggest that writing-based learning strengthens the involvement of this dorsal visuomotor pathway in reading, even when there was no explicit cue or demand of the visuomotor encoding.

### Left middle frontal gyrus

The uni-voxel results of learning and strategy in the left MFG were similar to the SPL: MFG showed increased responses after learning in both tasks, except that the effect for the pinyin group during VA_match task was only marginal. The pinyin + writing strategy resulted in a slightly stronger learning effect than the pinyin group only during the processing of orthographic-phonological mapping task. Unlike the SPL, the MFG was not found to display cross-participant consistent, strategy-specific multivoxel patterns during orthographic-only (Vl) processing. Thus, our interpretation of the results with caution was that writing-based learning seemed to increase the sensitivity of left MFG to print-to-sound matching, rather than orthographic processing per se Multiple roles have been proposed for MFG in logographic reading, such as visuospatial analysis (L. Liu et al., 2009; C.Y. Wu et al., 2012), orthography-semantics association (Siok et al., 2004; J. Wu et al., 2015), representing addressed phonology of Chinese words (Tan et al., 2005a; Booth et al., 2006; Kwok et al., 2019; A. Li et al., 2022), or encoding writing gestures (Nakamura et al., 2012). The present results showed that a sign of learning effect of the left MFG was observed during orthographic-to-phonological mapping process but not passive reading, which was consistent with insights from a previous meta-analysis that the activity in MFG is task dependent (Zhao et al., 2017). Because the joint presentation of written form and sound requires the processing of character identity, we speculate that the tendency of increased activity in left MFG reflects the development of knowledge about the sound-print association of a character. Because the learning effect in MFG was slightly amplified when participants learned to write, we speculate that the writing has provided additional assistance, which might be the increased orthographic awareness, for the learners to establish the sound-print association.

### Right fusiform gyrus

The responses in the right FG were distinct from the left SPL and MFG. Unlike the left FG, SPL, or MFG, R FG did not show an overall increase in the responses after learning. Moreover, for both the scrambled character and the learned real character, the R FG showed increased activations in the second scan, and this effect was reliably shown only in the pinyin group, only when the task was the unimodal visual processing (Fig. 5*C*). These findings suggest that the R FG is not specialized for recognizing scripts but for recognizing domain-general complex visual layout. A week of exposure to characters might increase its sensitivity to complex layout in general, but the multimodal pinyin-and-writing-based learning had downplayed the engagement of this area in character processing. Similarly, when the paradigm explicitly required multimodal knowledge of the characters, the R FG played less of a role as the other areas (MFG and SPL) took over the task of orthographic-phonological mapping.

One limitation of the study was that involving writing led to additional practice for that group of participants. The dilemma is that the control of workload means relatively less pinyin-based practice in the pinyin + writing group, the same amount of pinyin-based practice means more overall practice for the pinyin + writing group, whereas a writing-only learning procedure is unnatural and unlikely to be adopted in a realist situation for typically developed learners. We choose to examine the effect of additional writing practice, which results in unbalanced workload between groups. Future study is required to address whether the effect identified in the present work is writing-specific or just an effect of multimodal training, or even just an effect of more practice. It also remains an open question whether the activities of the so-called “logographic-specific” areas are also modulated by learning strategy if the target language is an alphabetic language. There has been evidence that a left IFG-premotor area (centered at [−42, 6, 20], close to the center of the MFG cluster used in the present study at [−46, 18, 28]) is sensitive to the correct moving trajectories of writing, and the effect seemed to be consistent in French words and Chinese characters (Nakamura et al., 2012). Such finding suggests that it might be the common processing of handwriting that results in the specialization of these seemingly language-specific areas. Future studies are required to directly investigate the effect of learning strategy on alphabetic languages.

Another limitation of the study was that the uni-voxel effects were only present at cluster level, indicating low spatial specificity. On the other hand, multivoxel patterns showed a strategy-related, cross-subject consistent patterns in the a priori regions. These findings might suggest a more distributed, i.e., less spatially specific, patterns for representing characters for the L2 learners.

Two groups of participants showed different learning effects in brain activations in the absence of behavioral differences. Previous study has observed neural differences in processing word and nonwords on adult L2 learners after several hours of learnings, when the behavioral performance was still at chance level (McLaughlin et al., 2004). Given the role of writing in Chinese learning, it is possible that neural group difference is a harbinger of overt behavioral differences.

Overall, the present study has revealed the emergence of logographic reading network after a week of learning in adult alphabetic reader’s brain. The learning effect in logographic-specific areas was not entirely dependent on, but modulated by the learning strategy. The present finding on group differences has suggested the additional effects of writing-based learning.

## Synthesis

Reviewing Editor: Anne Keitel, University of Dundee Decisions are customarily a result of the Reviewing Editor and the peer reviewers coming together and discussing their recommendations until a consensus is reached. When revisions are invited, a fact-based synthesis statement explaining their decision and outlining what is needed to prepare a revision will be listed below. The following reviewer(s) agreed to reveal their identity: Bo Yao.

The reviewers appreciated the revisions and thought that the manuscript was improved. However, there are a few issues to be addressed mainly regarding clarifications and justification of some analysis choices (reviewer #1) and providing a two-way ANOVA (reviewer #2).

I am attaching the unabridged reviewer comments below to allow a point-by-point reply.

Reviewer #1 - Advances the Field

The manuscript does advance the field by investigating how different strategies in learning Chinese characters (pinyin or pinyin+writing) may influence character learning in new learners who speak alphabetic languages. The study shows some of the logographic reading network is differentially engaged after different modes of learning although there was no behavioural difference in learning. The analyses now are appropriate and I applaud the addition of an MVPA which shows that the L SPL is particularly sensitive to different modes of learning. Although there are still outstanding questions (e.g., whether the differential activations are specific to writing-based learning or just increased / multimodal learning efforts), this dataset should make a nice contribution to the relevant reading literature.

Reviewer #1 - Statistics

yes for the most part but I have reservations on the neural specialisation aspect of it which I will detail later in my comments.

Reviewer #1 - Comments to the Authors

I thank the authors for revising the manuscript in accordance with both reviewers’ suggestions. I think the introduction has improved and the analyses are appropriate for the most parts (see my specific comments below). I applaud the addition of the MVPA which generates very interesting results (and raises more questions - which is not necessarily a bad thing). In the spirit of improving the manuscript further, I would like the authors to address some further comments:

Introduction

I think the authors’ points now come across more clearly in the introduction.

I do still think the literature review on different stages of character processing and which brain area is thought to do what should be elaborated. This is important and particularly helpful for readers who are not familiar with the Chinese reading literature to quickly grasp the state of the art.

Specifically in Line 59, you write ‘multiple components of character processing’. What are the components? I understand that in the study you focus on ‘orthographic processing’ and ‘orthographic-phonological mapping’. Because these two are not very well motivated in the introduction, they seem out of blue in Line 90-91. You need to highlight the roles of these two stages of processing in Chinese reading and their respective neural correlates as reported in the previous literature.

Line 78-80. I am not sure I understand what you mean by baseline because in the current study there wasn’t a contrast between Chinese speakers and a Chinese learner ‘baseline’. I guess you want to say that training effects on people who already speak Chinese are difficult to interpret because their brains are already ‘specialised’ for processing characters.? Please consider rephrasing.

Line 89. ‘artificial task’ sounds confusing. Perhaps ‘no explicit judgements/responses’?

Analysis

The authors now have addressed the learning effect problem. However there is another issue with the ‘neural specialisation’ claim. The manuscript tries to claim increased neural specialisation of XYZ as a result of learning but I couldn’t find clear definition of what neural specialisation means. My guess is that it means that these increased neural activations are specific to *real* character processing, and do not reflect, say, domain-general increases in familiarity with character-like visual patterns, etc.

If so here is the problem: the analyses on learned character (Vl) and scramble character processing (Vs) are done separately (see Line 406-411) - that is, there is a learning x group analysis on characters and the same analysis done separately on scrambled characters. The results are that there is a group effect on characters which is not statistically significant on scrambled characters. You can’t claim that the group effect is greater on characters than on scrambled characters without directly contrasting them. So to claim reading-specificity one needs to do a character (real vs. scrambled or Vl vs. Vs?) x learning (post vs. pre) x group (pinyin vs. pinyin+writing) ANOVA, any learning x group effects that survive the real vs. scrambled character contrasts can then be considered specialised for reading/learning. In a similar vein, to study neural specialisation for orthographic-phonological conversion, one needs to do a VA (VAmatch vs. VAmismatch) x learning (post vs. pre) x group (pinyin vs. pinyin+writing) ANOVA to examine the effects of learning x group that survive the VA_match vs. VA_mismatch contrasts (rather than do separate tests as in Figure 4B and 5B).

Line 288. voxel-wise cluster-forming threshold is usually p<.001 not p<.05. Could the authors justify the use of a more liberal threshold?

Line 297-298. Inconsistency of benchmark ROIs. A benchmark ROI (left FG) is included for right FG. Why is it needed specifically for FG? Perhaps you want to find out whether the character effects are lateralised or not? If that were the case, shouldn’t there be equivalent benchmark ROIs for left MFG and left SPL as well (i.e. R MFG, and R SPL)?

Line 328. I think this is a response to the other reviewer’s comment. I think it is good to rule out potential confounds. Contrasting bilinguals vs. monolinguals is one way to do it but why is it done in the MVPA only? Shouldn’t it be done in the whole-brain and ROI analyses too? A neater solution would be to increase bilingualism (binary-coded, mean-centred) as covariates in all analyses so there is no need to have a separate analysis for ruling it out. If the authors decided to do mono- vs. bi-lingual contrasts for all analyses separately, which would take up quite a lot of space for reporting a (probably) null effect, I think these should go into supplemental materials and are referenced in the main text.

Line 391. while other analyses used a voxel-wise p of 0.05, this particular analysis used 0.01. This is inconsistent and needs an explanation/justification.

Line 400-401. two-tailed tests please. How do you predetermine the direction of the group effects? Any references to back this up?

Line 404-405. How is decreased activation interpreted as ‘greater’ learning effect? I am not saying increased activation always means more or better. I am just pointing out that the other learning effects you report are based on increased activations. There is not clear logic as to what constitutes ‘greater’ or ‘worse’ learning, unless backed up by references?

Discussion

Line 439-442. Claiming ‘neural specialisation’ which might not be true. See first comment about analysis.

Line 449-451. Increased neural activations after learning does not necessarily suggest they reflect effective learning, unless the increased activations are specific to the learned stimuli (See first comment about analysis) or the changes in neural activations are modelled as a function of the size of the learning effect in each individual.

Line 469. What is a ‘constrained’ effect? Please clarify.

Line 520-521. How do you reconcile discrepant results between univoxel analyses and MPVA? It doesn’t make sense to read ‘writing-based learning seemed to increase the specialization of left MFG for print-to-sound matching’ when the previous sentence says it is ‘*not* found to display cross-participant consistent, strategy-specific multivoxel patterns during orthographic processing.’ Please discuss?

Line 549-550. Could you clarify how this is ‘arguably writing-specific’? The subsequent discussion goes on to explain how this may not be writing-specific and could reflect more effortful or multimodal learning. So what can we take home from this study then?

Another point I want the authors to discuss is the lack of group differences in the behavioural data, which they show is unlikely to be caused by a ceiling effect. The literature review says orthographical awareness/processing is more important in character learning and yet writing-based learning did not lead to better learning outcomes in the current study. It make intuitive sense that your brain responds differently to different learning experiences (or ‘leaving different marks’ as the authors put it). But should we care? Is this ‘neural marks’ meaningful in any way? I want to hear what the authors think about this and I am sure other readers do too.

Figures

Figure 5-1. would be helpful to name the clusters (not just the coordinates) so readers can quickly work out which ROIs are which.

Reviewer #2 - Advances the Field

This study leads to an understanding of how learning methods may shape functional organization.

Reviewer #2 - Statistics

Needs to do two-way ANOVA

Reviewer #2 - Comments to the Authors

In this revised manuscript, the authors have addressed most of the concerns I had with the previous submission. In general, I found the manuscript to be in good shape, except that I still have a concern about fMRI data analysis.

The authors explained that they modelled the within-subject pre- and post-training effects in first level analysis, whereas the between-subject training conditions in the second level analysis. I found this method far from convincing, since the pre and post training scans were acquired in separate runs on different days. A more ideal way would be to model all the conditions in the second level, using two-way ANOVA.

## References

[B1] Bi Y, Han Z, Zhang Y (2009) Reading does not depend on writing, even in Chinese. Neuropsychologia 47:1193–1199. 10.1016/j.neuropsychologia.2008.11.006 19056407

[B2] Bolger DJ, Perfetti CA, Schneider W (2005) Cross-cultural effect on the brain revisited: universal structures plus writing system variation. Hum Brain Mapp 25:92–104. 10.1002/hbm.20124 15846818 PMC6871743

[B3] Booth JR, Lu D, Burman DD, Chou TL, Jin Z, Peng DL, Zhang L, Ding GS, Deng Y, Liu L (2006) Specialization of phonological and semantic processing in Chinese word reading. Brain Res 1071:197–207. 10.1016/j.brainres.2005.11.097 16427033 PMC2626184

[B4] Brysbaert M, New B (2009) Moving beyond Kucera and Francis: a critical evaluation of current word frequency norms and the introduction of a new and improved word frequency measure for American English. Behav Res Methods 41:977–990. 10.3758/BRM.41.4.977 19897807

[B5] Cai Q, Brysbaert M (2010) SUBTLEX-CH: Chinese word and character frequencies based on film subtitles. PLoS One 5:e10729. 10.1371/journal.pone.0010729 20532192 PMC2880003

[B6] Cao F, Perfetti CA (2016) Neural signatures of the reading-writing connection: greater involvement of writing in Chinese reading than English reading. PLoS One 11:e0168414. 10.1371/journal.pone.0168414 27992505 PMC5161366

[B7] Cao F, Vu M, Lung CD, Lawrence JM, Harris LN, Guan Q, Xu Y, Perfetti CA (2013) Writing affects the brain network of reading in Chinese: a functional magnetic resonance imaging study. Hum Brain Mapp 34:1670–1684. 10.1002/hbm.22017 22378588 PMC6870511

[B8] Cao F, Sussman BL, Rios V, Yan X, Wang Z, Spray GJ, Mack RM (2017) Different mechanisms in learning different second languages: evidence from English speakers learning Chinese and Spanish. Neuroimage 148:284–295. 10.1016/j.neuroimage.2017.01.042 28110086

[B9] Cohen L, Lehéricy S, Chochon F, Lemer C, Rivaud S, Dehaene S (2002) Language‐specific tuning of visual cortex? Functional properties of the visual word form area. Brain 125:1054–1069. 10.1093/brain/awf094 11960895

[B10] Dehaene S, Cohen L (2007) Cultural recycling of cortical maps. Neuron 56:384–398. 10.1016/j.neuron.2007.10.004 17964253

[B11] Dehaene S, Naccache L, Cohen L, Le Bihan D, Mangin J-F, Poline JB, Rivière D (2001) Cerebral mechanisms of word masking and unconscious repetition priming. Nat Neurosci 4:752–758. 10.1038/89551 11426233

[B12] Dehaene S, Pegado F, Braga LW, Ventura P, Filho GN, Jobert A, Dehaene-Lambertz G, Kolinsky R, Morais J, Cohen L (2010) How learning to read changes the cortical networks for vision and language. Science 330:1359–1364. 10.1126/science.1194140 21071632

[B13] Dong J, Li A, Chen C, Qu J, Jiang N, Sun Y, Hu L, Mei L (2021) Language distance in orthographic transparency affects cross-language pattern similarity between native and non-native languages. Hum Brain Mapp 42:893–907. 10.1002/hbm.25266 33112483 PMC7856648

[B14] Eden GF, Moats L (2002) The role of neuroscience in the remediation of students with dyslexia. Nat Neurosci 5:1080–1084. 10.1038/nn946 12403991

[B15] Feng XX, Altarelli I, Monzalvo K, Ding GS, Ramus F, Shu H, Dehaene S, Meng XZ, Dehaene-Lambertz G (2020) A universal reading network and its modulation by writing system and reading ability in French and Chinese children. Elife 9:e54591. 10.7554/eLife.54591 33118931 PMC7669264

[B16] Gollan TH, Weissberger GH, Runnqvist E, Montoya RI, Cera CM (2012) Self-ratings of spoken language dominance: a multilingual naming test (MINT) and preliminary norms for young and aging Spanish-English bilinguals. Biling (Camb Engl) 15:594–615. 10.1017/S1366728911000332 25364296 PMC4212892

[B17] Haspelmath M, Dryer MS, Gil D, Comrie B (2008) World Atlas of Language Structures [www document]. Munich Max Planck Digit Libr Available at http://wals.info.

[B18] Hu W, Lee HL, Zhang Q, Liu T, Geng LB, Seghier ML, Shakeshaft C, Twomey T, Green DW, Yang YM, Price CJ (2010) Developmental dyslexia in Chinese and English populations: dissociating the effect of dyslexia from language differences. Brain 133:1694–1706. 10.1093/brain/awq106 20488886 PMC2877905

[B19] Keuleers E, Stevens M, Mandera P, Brysbaert M (2015) Word knowledge in the crowd: measuring vocabulary size and word prevalence in a massive online experiment. Q J Exp Psychol (Hove) 68:1665–1692. 10.1080/17470218.2015.1022560 25715025

[B20] Kim SY, Qi T, Feng X, Ding G, Liu L, Cao F (2016) How does language distance between L1 and L2 affect the L2 brain network? An fMRI study of Korean–Chinese–English trilinguals. Neuroimage 129:25–39. 10.1016/j.neuroimage.2015.11.068 26673115

[B21] Kochunov P, Fox P, Lancaster J, Tan LH, Amunts K, Zilles K, Mazziotta J, Gao JH (2003) Localized morphological brain differences between English-speaking Caucasians and Chinese-speaking Asians: new evidence of anatomical plasticity. Neuroreport 14:961–964. 12802183 10.1097/01.wnr.0000075417.59944.00

[B22] Kuo WJ, Yeh TC, Lee JR, Chen LF, Lee PL, Chen SS, Ho LT, Hung DL, Tzeng OJL, Hsieh JC (2004) Orthographic and phonological processing of Chinese characters: an fMRI study. Neuroimage 21:1721–1731. 10.1016/j.neuroimage.2003.12.007 15050593

[B23] Kwok VPY, Matthews S, Yakpo K, Tan LH (2019) Neural correlates and functional connectivity of lexical tone processing in reading. Brain Lang 196:104662. 10.1016/j.bandl.2019.104662 31352216

[B24] Lagarrigue A, Longcamp M, Anton JL, Nazarian B, Prévot L, Velay J-L, Cao F, Frenck-Mestre C (2017) Activation of writing-specific brain regions when reading Chinese as a second language. Effects of training modality and transfer to novel characters. Neuropsychologia 97:83–97. 10.1016/j.neuropsychologia.2017.01.026 28131811

[B25] Lewis MP, Simons GF, Fennig C (2014) Ethnologue: languages of the world, Ed 17. Dallas: SIL International.

[B26] Li A, Yang R, Qu J, Dong J, Gu L, Mei L (2022) Neural representation of phonological information during Chinese character reading. Hum Brain Mapp 43:4013–4029. 35545935 10.1002/hbm.25900 PMC9374885

[B27] Li P, Zhang F, Tsai E, Puls B (2014) Language history questionnaire (LHQ 2.0): a new dynamic web-based research tool. Bilingualism 17:673–680. 10.1017/S1366728913000606

[B28] Liu L, Deng X, Peng D, Cao F, Ding G, Jin Z, Zeng Y, Li K, Zhu L, Fan N, Deng Y, Bolger DJ, Booth JR (2009) Modality- and task-specific brain regions involved in Chinese lexical processing. J Cogn Neurosci 21:1473–1487. 10.1162/jocn.2009.21141 18823229 PMC3277453

[B29] Liu Y, Dunlap S, Fiez J, Perfetti C (2007a) Evidence for neural accommodation to a writing system following learning. Hum Brain Mapp 28:1223–1234. 10.1002/hbm.20356 17274024 PMC6871335

[B30] Liu Y, Shu H, Li P (2007b) Word naming and psycholinguistic norms: Chinese. Behav Res Methods 39:192–198. 10.3758/bf03193147 17695344

[B31] Ma JW, Wu YJ, Sun T, Cai L, Fan XX, Li XH (2020) Neural substrates of bilingual processing in a logographic writing system: an fMRI study in Chinese Cantonese-Mandarin bilinguals. BRAIN Res 1738. 10.1016/j.brainres.2020.146794 32234428

[B32] Maisog JM, Einbinder ER, Flowers DL, Turkeltaub PE, Eden GF (2008) A meta-analysis of functional neuroimaging studies of dyslexia. Ann N Y Acad Sci 1145:237–259. 10.1196/annals.1416.024 19076401

[B33] McArthur G, Sheehan Y, Badcock NA, Francis DA, Wang H-C, Kohnen S, Banales E, Anandakumar T, Marinus E, Castles A (2018) Phonics training for English-speaking poor readers. Cochrane database Syst Rev 11:CD009115. 10.1002/14651858.CD009115.pub3 30480759 PMC6517252

[B34] McLaughlin J, Osterhout L, Kim A (2004) Neural correlates of second-language word learning: minimal instruction produces rapid change. Nat Neurosci 7:703–704. 10.1038/nn1264 15195094

[B35] Meade G (2020) The role of phonology during visual word learning in adults: an integrative review. Psychon Bull Rev 27:15–23. 10.3758/s13423-019-01647-0 31422528

[B36] Nakamura K, Kuo WJ, Pegado F, Cohen L, Tzeng OJL, Dehaene S (2012) Universal brain systems for recognizing word shapes and handwriting gestures during reading. Proc Natl Acad Sci U S A 109:20762–20767. 10.1073/pnas.1217749109 23184998 PMC3528608

[B37] Nelson JR, Liu Y, Fiez J, Perfetti CA (2009) Assimilation and accommodation patterns in ventral occipitotemporal cortex in learning a second writing system. Hum Brain Mapp 30:810–820. 10.1002/hbm.20551 18381767 PMC5283558

[B38] Oldfield RC (1971) The assessment and analysis of handedness: the Edinburgh inventory. Neuropsychologia 9:97–113. 10.1016/0028-3932(71)90067-4 5146491

[B39] Paulesu E, McCrory E, Fazio F, Menoncello L, Brunswick N, Cappa SF, Cotelli M, Cossu G, Corte F, Lorusso M, Pesenti S, Gallagher A, Perani D, Price C, Frith CD, Frith U (2000) A cultural effect on brain function. Nat Neurosci 3:91–96. 10.1038/71163 10607401

[B40] Raven J (2017) Raven progressive matrices. In: Handbook of nonverbal assessment (RS McCallum, ed). Boston: Springer.

[B41] Richlan F, Kronbichler M, Wimmer H (2009) Functional abnormalities in the dyslexic brain: a quantitative meta-analysis of neuroimaging studies. Hum Brain Mapp 30:3299–3308. 10.1002/hbm.20752 19288465 PMC2989182

[B42] Rueckl JG, Paz-Alonso PM, Molfese PJ, Kuo WJ, Bick A, Frost SJ, Hancock R, Wu DH, Mencl WE, Duñabeitia JA, Lee JR, Oliver M, Zevin JD, Hoeft F, Carreiras M, Tzeng OJL, Pugh KR, Frost R (2015) Universal brain signature of proficient reading: evidence from four contrasting languages. Proc Natl Acad Sci U S A 112:15510–15515. 10.1073/pnas.1509321112 26621710 PMC4687557

[B43] Siok WT, Perfetti CA, Jin Z, Tan LH (2004) Biological abnormality of impaired reading is constrained by culture. Nature 431:71–76. 10.1038/nature02865 15343334

[B44] Siok WT, Spinks JA, Jin Z, Tan LH (2009) Developmental dyslexia is characterized by the co-existence of visuospatial and phonological disorders in Chinese children. Curr Biol 19:R890–R892. 10.1016/j.cub.2009.08.014 19825347

[B45] Skirgård H, Roberts SG, Yencken L (2017) Why are some languages confused for others? Investigating data from the great language game. PLoS One 12:e0165934. 10.1371/journal.pone.0165934 28379970 PMC5381764

[B46] Stone JM, Towse JN (2015) A working memory test battery: Java-based collection of seven working memory tasks. J Open Res Softw 3:e5.

[B47] Tan LH, Liu HL, Perfetti CA, Spinks JA, Fox PT, Gao JH (2001) The neural system underlying Chinese logograph reading. Neuroimage 13:836–846. 10.1006/nimg.2001.0749 11304080

[B48] Tan LH, Laird AR, Li K, Fox PT (2005a) Neuroanatomical correlates of phonological processing of Chinese characters and alphabetic words: a meta-analysis. Hum Brain Mapp 25:83–91. 10.1002/hbm.20134 15846817 PMC6871734

[B49] Tan LH, Spinks JA, Eden GF, Perfetti CA, Siok WT (2005b) Reading depends on writing, in Chinese. Proc Natl Acad Sci U S A 102:8781–8785. 10.1073/pnas.0503523102 15939871 PMC1150863

[B50] Temple E, Deutsch GK, Poldrack RA, Miller SL, Tallal P, Merzenich MM, Gabrieli JDE (2003) Neural deficits in children with dyslexia ameliorated by behavioral remediation: evidence from functional MRI. Proc Natl Acad Sci U S A 100:2860–2865. 10.1073/pnas.0030098100 12604786 PMC151431

[B51] Transvaal Education Department (1987) One-minute reading test.

[B52] Vellutino FR, Fletcher JM, Snowling MJ, Scanlon DM (2004) Specific reading disability (dyslexia): what have we learned in the past four decades? J Child Psychol Psychiatry 45:2–40. 10.1046/j.0021-9630.2003.00305.x 14959801

[B53] Verhoeven L, Perfetti C (2021) Universals in learning to read across languages and writing systems. Sci Stud Read 26:150–164.

[B54] Wagner RK, Torgesen JK, Rashotte CA, Hecht SA, Barker TA, Burgess SR, Donahue J, Garon T (1997) Changing relations between phonological processing abilities and word-level reading as children develop from beginning to skilled readers: a 5-year longitudinal study. Dev Psychol 33:468–479. 10.1037//0012-1649.33.3.468 9149925

[B55] Wiley RW, Rapp B (2021) The effects of handwriting experience on literacy learning. Psychol Sci 32:1086–1103. 10.1177/0956797621993111 34184564 PMC8641140

[B56] Wong YK, Zhou YL (2022) Effects of metalinguistic awareness on Chinese as a second language spelling through the mediation of reading and copying. Read Writ 35:853–875. 10.1007/s11145-021-10167-0

[B57] Wu CY, Ho MHR, Chen SHA (2012) A meta-analysis of fMRI studies on Chinese orthographic, phonological, and semantic processing. Neuroimage 63:381–391. 10.1016/j.neuroimage.2012.06.047 22759996

[B58] Wu J, Lu J, Zhang H, Zhang J, Yao C, Zhuang D, Qiu T, Guo Q, Hu X, Mao Y, Zhou L (2015) Direct evidence from intraoperative electrocortical stimulation indicates shared and distinct speech production center between Chinese and English languages. Hum Brain Mapp 36:4972–4985. 10.1002/hbm.22991 26351094 PMC6869327

[B59] Ye YY, Yan MG, Ruan YJ, Catherine M, Yeung CF (2021) Literacy learning in early Chinese-English bilinguals: the role of pure copying skill. Early Child Res Q 55:263–274. 10.1016/j.ecresq.2020.12.004

[B60] Zhai M, Fischer-Baum S (2019) Exploring the effects of knowledge of writing on reading Chinese characters in skilled readers. J Exp Psychol Learn Mem Cogn 45:724–731. 10.1037/xlm0000604 29999402 PMC6330147

[B61] Zhao R, Fan R, Liu MX, Wang XJ, Yang JF (2017) Rethinking the function of brain regions for reading Chinese characters in a meta-analysis of fMRI studies. J Neurolinguistics 44:120–133. 10.1016/j.jneuroling.2017.04.001

[B62] Zhou W, Xia ZC, Bi YC, Shu H (2015) Altered connectivity of the dorsal and ventral visual regions in dyslexic children: a resting-state fMRI study. Front Hum Neurosci 9:495. 26441595 10.3389/fnhum.2015.00495 PMC4564758

[B63] Ziegler JC, Goswami U (2005) Reading acquisition, developmental dyslexia, and skilled reading across languages: a psycholinguistic grain size theory. Psychol Bull 131:3–29. 10.1037/0033-2909.131.1.3 15631549

