# Supplementary figures and images for: How Characters Are Learned Leaves Its Mark on the Neural Substrates of Chinese Reading
Source: eNeuro. 2022 Dec 21;9(6):ENEURO.0111-22.2022. doi: 10.1523/ENEURO.0111-22.2022 (PMC9787807; doi:10.1523/ENEURO.0111-22.2022)

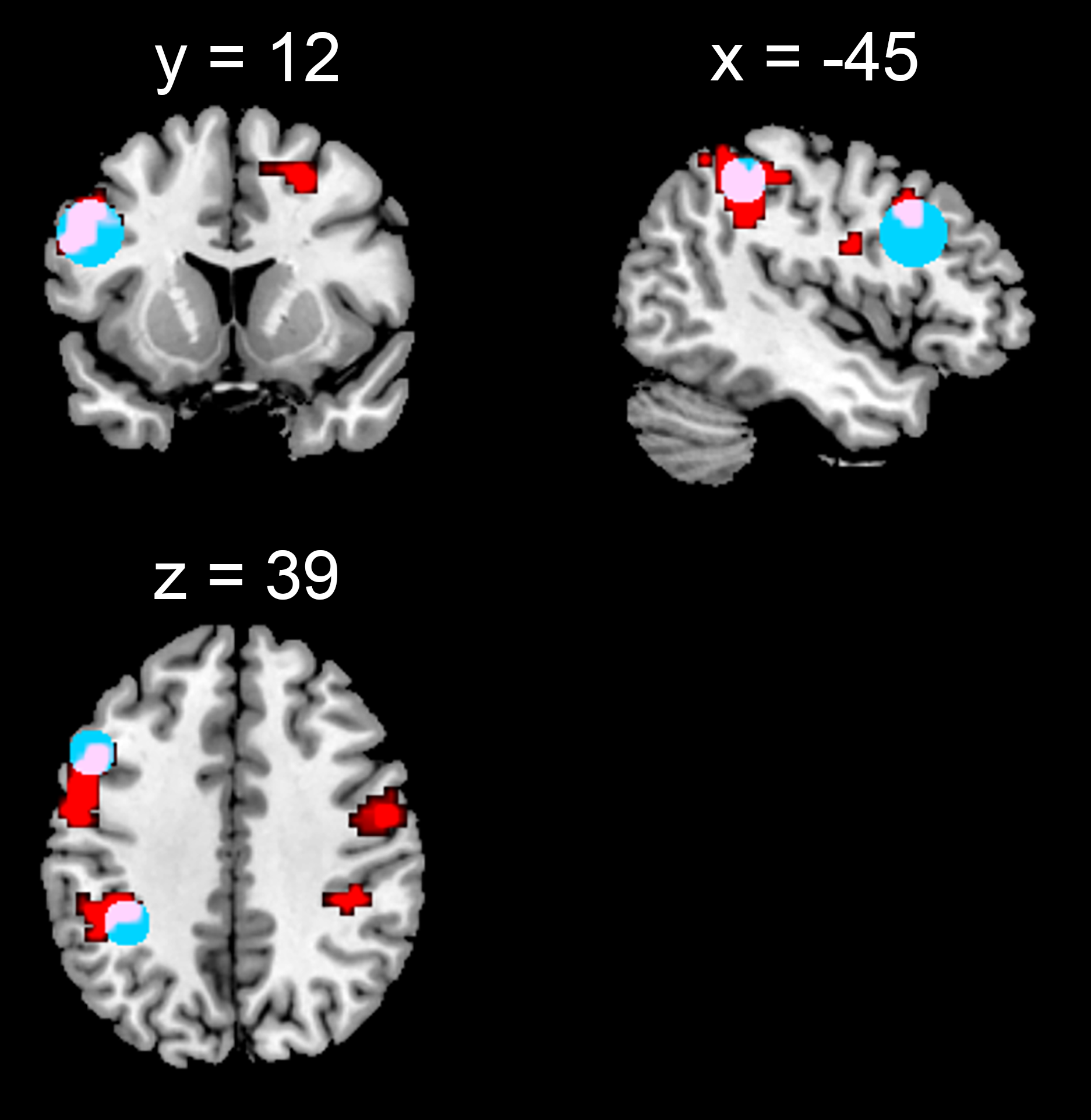

Supplement: Extended Data Figure 5-2 — Overlap of a priori ROIs (in blue) and results of the whole-brain analysis of strategy effect (pinyin + writing > pinyin; in red). The overlapped areas are shown in pale pink. Download Figure 5-2, TIF file. [file enu-eN-NWR-0111-22-s03.tif]

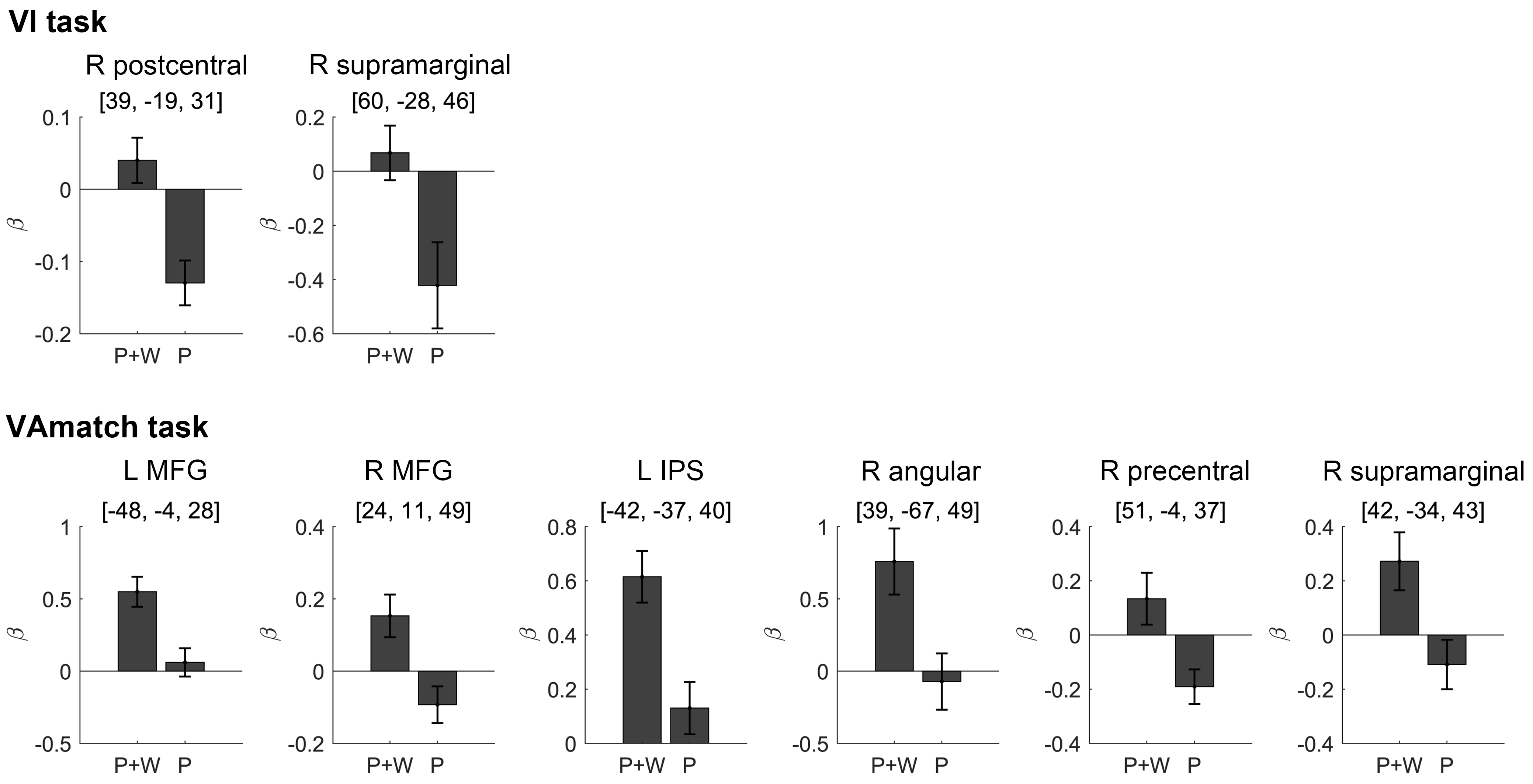

Supplement: Extended Data Figure 5-1 — Post hoc analysis of the effect of strategy on the learning effect. Mean and SE of each cluster in the two tasks for the pinyin (P) and pinyin + writing (P + W) groups was plotted. Download Figure 5-1, TIF file. [file enu-eN-NWR-0111-22-s02.tif]
