# Supplementary material for: How Characters Are Learned Leaves Its Mark on the Neural Substrates of Chinese Reading
Source: eNeuro. 2022 Dec 21;9(6):ENEURO.0111-22.2022. doi: 10.1523/ENEURO.0111-22.2022 (PMC9787807; doi:10.1523/ENEURO.0111-22.2022)
Supplement: Extended Data Figure 5-3 — Accuracy of classifying participant’s language background. Download Figure 5-3, DOC file. [file enu-eN-NWR-0111-22-s04.doc]

**Extended Data**

**Figure 5-3.** Accuracy of classifying participant’s language background.

|  | Visual | Visual-auditory |
| --- | --- | --- |
| L SPL | 0.43 | 0.43 |
| L MFG | 0.53 | 0.37 |
| R FG | 0.33 | 0.57 |
| L FG | 0.40 | 0.57 |


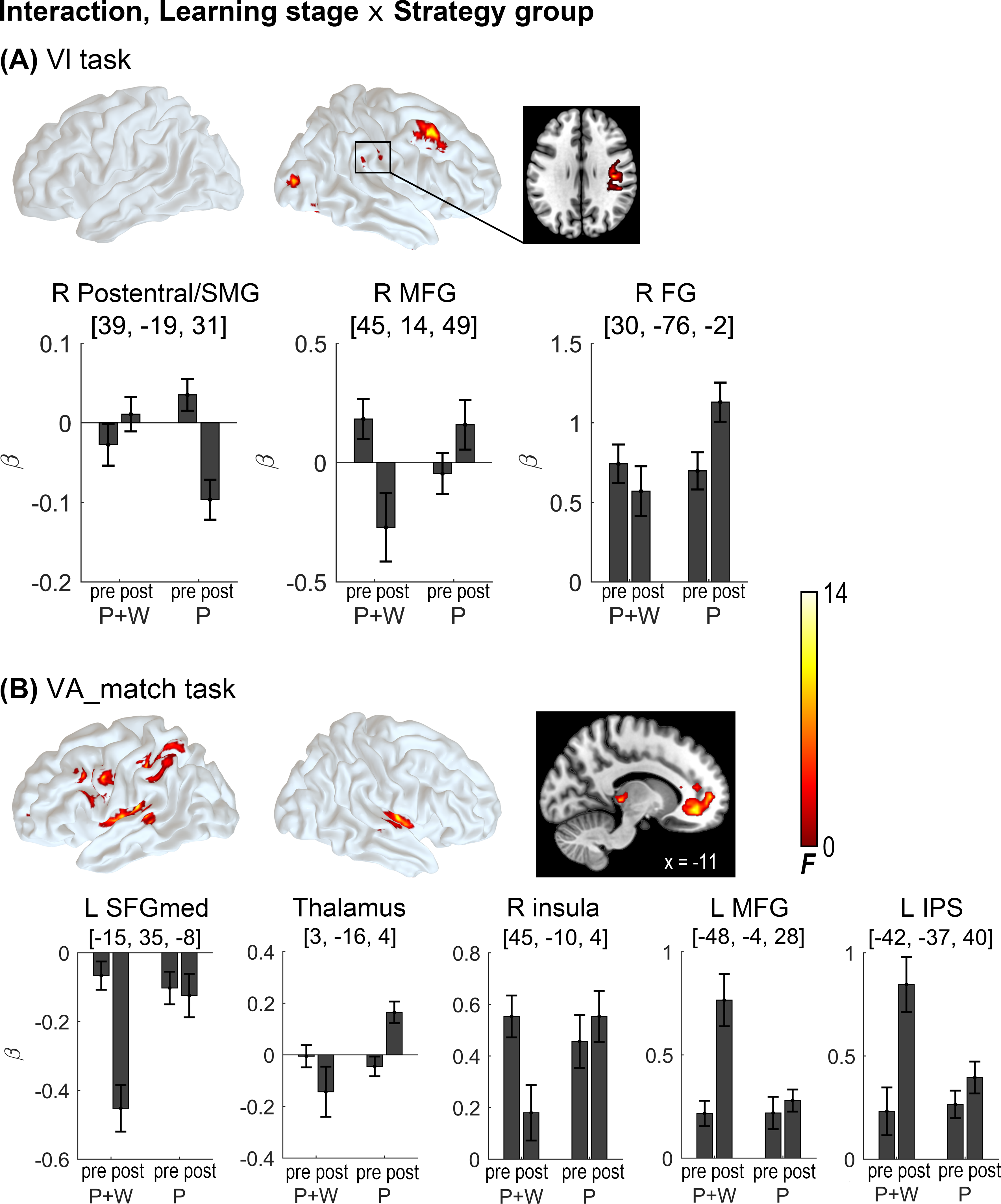


**Figure 5-4.** ANOVAs on the learning stage × strategy interaction in visual (A) and visual-auditory (B) tasks.

**Figure 5-5.** Clusters presenting the learning stage × strategy interaction effect based on ANOVAs.

| Pinyin + writing vs. pinyin | H | K | MNI coordinate | | | F |
| --- | --- | --- | --- | --- | --- | --- |
|  |  | (voxels) | x | y | z |  |
| **Visual** |  |  |  |  |  |  |
| Postcentral gyrus | R | 98 | 39 | -19 | 31 | 11.61 |
| Supramarginal gyrus |  |  | 36 | -31 | 31 | 9.10 |
| Supramarginal gyrus |  |  | 45 | -34 | 31 | 7.58 |
| Middle frontal gyrus | R | 106 | 45 | 14 | 49 | 11.81 |
| Middle frontal gyrus |  |  | 39 | 5 | 46 | 7.92 |
| Middle frontal gyrus |  |  | 45 | 23 | 37 | 7.12 |
| Fusiform gyrus | R | 130 | 30 | -76 | -2 | 10.23 |
| Middle occipital gyrus |  |  | 30 | -82 | 7 | 9.60 |
| Inferior occipital gyrus |  |  | 39 | -76 | -11 | 7.83 |
|  |  |  |  |  |  |  |
| **Visual-auditory** |  |  |  |  |  |  |
| Superior medial frontal lobe | L | 351 | -15 | 35 | -8 | 14.32 |
| Superior medial frontal lobe |  |  | -9 | 53 | 4 | 12.35 |
| Anterior cingulate cortex |  |  | -15 | 41 | -2 | 12.20 |
| Thalamus | L | 145 | 3 | -16 | 4 | 12.59 |
| Thalamus |  |  | -12 | -31 | 1 | 11.78 |
| Thalamus |  |  | 21 | -28 | 7 | 11.09 |
| Insula | R | 122 | 45 | -10 | 4 | 11.53 |
| Insula |  |  | 45 | -4 | -2 | 9.86 |
| Superior temporal gyrus |  |  | 54 | -16 | 1 | 8.36 |
| Precentral gyrus | L | 152 | -48 | -4 | 28 | 10.78 |
| Middle frontal gyrus |  |  | -51 | 8 | 37 | 10.58 |
| Intraparietal sulcus | L | 135 | -42 | -37 | 40 | 9.06 |
| Superior parietal lobule |  |  | -42 | -52 | 58 | 5.71 |
